# Density and seasonal dynamics of *Bemisia tabaci* and its predators in different agricultural landscapes in South China

**DOI:** 10.3389/fpls.2022.928634

**Published:** 2022-09-02

**Authors:** Ming-Jiang Li, Shao-Wu Yang, Guo-Hua Chen, Wen-Jun Dou, Hao-Pei Shang, Xiao-Ming Zhang

**Affiliations:** ^1^National Key Laboratory for Conservation and Utilization of Biological Resources in Yunnan, College of Plant Protection, Yunnan Agricultural University, Kunming, China; ^2^Yunnan Expo Horticulture Co., Ltd., Kunming, China

**Keywords:** *Bemisia tabaci*, agriculture landscapes, population dynamics, dominance, temporal niche, predators

## Abstract

*Bemisia tabaci* is the main pest of agriculture in many regions of the world. The resistance of whitefly to pesticides has increased as a consequence of the continuous irrational use of wide-spectrum pesticides. Thus, pesticides are no longer always effective as a long-term control method. The agricultural landscape can affect the occurrence of an insect population. The objective of this study was to clarify the occurrence of whitefly and its predators in tomato fields in different agricultural landscapes. Different landscapes are classified into urban, flower, water, and mountain landscapes by the principal component analysis method. In 2018–2019, whitefly had the longest main activity period and the lowest density in the flower landscape. The water landscape helped to maintain the highest densities of whitefly during the main activity period. Nine species of predators were sampled, and *Nesidiocoris tenuis*, *Chrysoperla sinica*, *Menochilus sexmaculata*, and *Harmonia axyridis* were the dominant species throughout the sampling season in both years. During the main activity period, *N. tenuis* had the highest density in all sampled landscapes. The density of the dominant predators was the highest in the flower landscape, and each natural predator had the largest temporal niche width in the 2-year sampling period. *Bemisia tabaci*, *N. tenuis*, and *M. sexmaculata* were highly synchronized temporally. The flower landscape showed satisfactory results in suppressing whitefly. Increasing the proportion of flowering plants and increasing the diversity of plant crops in the agricultural landscape can effectively reduce the densities of whitefly during an outbreak.

## Introduction

The whitefly *Bemisia tabaci* (Gennadius) (Hemiptera: Aleyrodidae) is the main pest of economic agriculture in several countries and regions of the world (Pan et al., 2021). It is distributed worldwide except in Antarctica and has caused serious economic losses to global agricultural production (Barro et al., 2011). The host plant species of whitefly are very extensive (Barro et al., 2011; Jiang, 2020). In China, plants that are most often damaged include *Solanum lycopersicum* Miller (Tubiflorae: Solanaceae), *Brassica oleracea* L. (Brassicales: Brassicaceae), *Cucumis sativus* L. (Cucurbitales: Cucurbitaceae), *Gossypium* spp. (Malvales: Malvaceae), and other economic crops (Zhang et al., 2014a). Moreover, whitefly directly sucks plant sap by piercing-sucking mouthparts and secretes honeydew, which induces sooty blotch and seriously affects plant photosynthesis (Zhang et al., 2014b). However, ingestion of plant sap by whiteflies can also indirectly transmit several plant viruses, causing far more economic losses than those from direct feeding (Muniyappa and Veeresh, 1984; Li et al., 2010; Zhang et al., 2020b). Tomato is an important edible vegetable that is widely cultivated in China (Miao, 2017). In 2018, the tomato planting area in Yunnan province was approximately 35,600 hm^2^, divided mainly into two summer and winter planting seasons. The main method of planting tomatoes in Yunnan province is open field cultivation, and the greenhouse cultivation area accounts for 33.3% of the total planting area (Zhao et al., 2019). The volume of tomato exports in Yunnan province accounts for 37% of the China export volume, and the yield per unit area in Kunming city is the highest (Zhao et al., 2019).

Chemical control methods are widely used in the control of whiteflies because of their advantages of quick action, low cost, and easy operation. However, with the long-term unregulated application of chemical pesticides, the resistance of the whitefly has increased, and several chemical pesticides have been unable to control it (Yang et al., 2014). Biological control has the advantages of economical application and environmental safety (Suzanne, 1982). Due to the increasing resistance of whitefly, the use of biological control methods has become the main trend in the integrated management of whiteflies (Lenteren et al., 2018, 2020).

Natural enemies of whiteflies are abundant. There are 128 species of predators and more than 90 species of parasitoids of whiteflies in the world (Gerling et al., 2001; Li et al., 2011). Currently, 109 species of predators and 59 species of parasitoids of whitefly have been reported in China (Li et al., 2011; Dou et al., 2020a). The biological control of the whitefly has been studied and applied worldwide for more than 40 years (Ren et al., 2001). There are about 13,000 species of insects in Yunnan province, China, accounting for approximately 25.5% of the country (Liu and Xia, 2017), and the natural enemy resources are highly rich (Dou et al., 2020a). At present, only two parasitoids of *Aphidius gifuensis* Ashmead (Hymenoptera: Aphidiidae) and *Scleroderma* spp. (Hymenoptera: Bethylidae) in Yunnan province are widely used in agricultural and forest production (Liu and Xia, 2017). However, there are few reports on pest control in the tomato field by adjusting the landscape layout. The efficacy of the control of whitefly by natural enemies is affected by several factors. The landscape pattern of the agroecosystem, especially the composition and configuration of crop and non-crop habitats, can affect the population density of pests and their natural enemies, thus, affecting the efficacy of control by natural enemies (Jonsson et al., 2012; Veres et al., 2013). Agroecosystems are unstable artificial ecosystems. In terms of habitats, it includes crop habitats and surrounding non-farming habitats (You et al., 2004). Crop habitats provide the main breeding ground for pests and their natural enemies. In contrast, non-crop habitats can be used as sites for pests and natural enemies to find alternative hosts and escape adverse environmental conditions (Chaplin-Kramer et al., 2011). At present, the widespread planting pattern is characterized by the increase of intensive production mode, which leads to the single structure of farmland landscape and greatly reduces the biodiversity of farmland landscape (Tscharntke et al., 2002). Recent studies have shown that the pattern of distribution of biodiversity in farmland is affected by the structure of the surrounding landscape (Zhang, 2017). The different agricultural landscape has formed a unique pattern of community planting, and some places have even gradually presented a patchy pattern (Yu et al., 1996). Generally, the non-crop habitat alters the biodiversity of the environment. Non-crop habitats may host several natural enemies, which could have an important role in pest control (Liu et al., 2000). However, with the advent of the intensive production mode, the interspecific relationship of insects has changed, and the control effect of natural enemies has been affected (Bianchi et al., 2006). Some studies analyzed the effects of farmland landscape patterns and pesticides use on ladybugs in cotton fields and demonstrated that *Zea mays* L. (Poales: Poaceae) and grassland habitats were more conducive to the occurrence of ladybugs in wheat fields (Zhou et al., 2014). The influence of forest cover in different farmland landscapes on ladybug diversity was studied. A large area of woodland in the agricultural landscape was observed to be conducive to ladybug migration to wheat fields (Zhao K. D. et al., 2015). The composition of farmland and its surrounding non-crop habitats plays an important role in the occurrence and migration of insects. By changing the vegetation composition of non-crop habitats in the farmland landscape, the control efficiency of natural enemies can be improved (You et al., 2004). A study of the effects of different agricultural landscapes on the natural enemies of *Pyrausta nubilalis* (Hubern) (Lepidoptera: Pyralidae) demonstrated that natural enemies of *P. nubilalis* gathered the most when the proportion of non-cultivated habitats, especially forest land, villages, and grasslands, was 20–30% (Bian et al., 2019). Research on the effects of agricultural landscape structure on the protection of natural enemies and pest control, the composition of non-crop habitats, and the combination of crop and non-crop habitats have emphasized the importance of protecting the diversity of natural enemies (Bianchi et al., 2006). In summary, crop habitat and non-crop habitat compositions and their area ratio in the farmland landscape significantly affect insect richness, and a reasonable landscape layout can promote the control of pests by natural enemy insects.

In this study, the occurrence of whitefly in tomato fields of different agricultural landscapes in Kunming city, Yunnan province, as well as the resultant effects of its predators, were studied. The study also aimed to explore the effects of different agricultural landscapes on the populations of whitefly and their predators. The results could provide a theoretical basis for the ecological regulation of the whitefly and the ecological planning of the farmland landscape.

## Materials and methods

### Site design

This study was replicated at 12 plot site fields on a landscape diversity gradient in Kunming city, Yunnan province, south China of tomato growing areas during the second half of 2018 and 2019. The climate type belongs to the subtropical monsoon climate. The area of each site is about 800 square meters (20 m × 40 m), located more than 5 km apart from each replicate site. The tomatoes are planted in an open field in all plots. The tomato cultivar is “Zhongyan TV1” (Beijing Zhongyan Yinong Seedling Co., Ltd.). The tomato varieties and the density of the planting are consistent in all tomato planting plots. The sampling started 1 week after tomato planting. The pesticides were not sprayed during the investigation, and we compensated farmers for their losses at all sites. Detailed agronomic parameters are shown in Table 1.

**TABLE 1:** Agronomic parameters of summer tomato fields in four different agricultural landscapes both in 2018 and 2019.

Landscape patterns	Years	Planting date	Removal date	Cultivar	Planting type	Plant spacing	Row spacing	Chemical treatments	Pruning scheme
Urban	2018	7–10	11–25	Zhongyan TV1	Open field	30 cm	50 cm	No	Double stem pruning
	2019	7–11	10–30	Zhongyan TV1	Open field	30 cm	50 cm	No	Double stem pruning
Flower	2018	6–13	10–30	Zhongyan TV1	Open field	30 cm	50 cm	No	Double stem pruning
	2019	6–27	11–12	Zhongyan TV1	Open field	30 cm	50 cm	No	Double stem pruning
Water	2018	6-26	10–30	Zhongyan TV1	Open field	30 cm	50 cm	No	Double stem pruning
	2019	7–11	10–30	Zhongyan TV1	Open field	30 cm	50 cm	No	Double stem pruning
Mountain	2018	7–10	11–13	Zhongyan TV1	Open field	30 cm	50 cm	No	Double stem pruning
	2019	6–27	10–20	Zhongyan TV1	Open field	30 cm	50 cm	No	Double stem pruning

Urban: Tomato fields of an agricultural landscape dominated by urban, flower: tomato fields of an agricultural landscape dominated by flower, water: tomato fields of an agricultural landscape dominated by water, and mountain: tomato fields of an agricultural landscape dominated by a mountain. The same for Tables 4–7.

### Sampling

Summer tomatoes in 2018 were sampled every 10 days from June 20 to the end of the growing season (November 25) in 2018. The summer tomatoes in 2019 started on July 4 and continued every 10 days until the end of the tomato growing season (November 12) in 2019.

Five random positions were selected in each sample plot site, and the five closest tomato plants were randomly selected and sampled from each position (avoiding the plants closest to any edge to minimize edge effects). From each tomato plant, five leaves of similar age were examined in the upper, middle, and lower positions, resulting in a total of 375 leaves being monitored per sample plot (Zhang et al., 2020b). To observe the number of adult *B. tabaci* and their predators, the unidentified individuals were brought back to the laboratory for identification. The leaves were then removed, placed in a Ziplock bag for marking, and brought back to the laboratory. The number of whitefly nymphs, namely, first, second, third, and fourth instar nymphs, was observed and recorded under the stereomicroscope (OLYMPUS, SZ51). Finally, the leaf area was recorded using a transparent graph paper placed on the leaf, and standardized density data (no. of individuals per 100 cm^2^ leaf surface) were calculated (Zhang et al., 2020b).

### Describing seasonal activity

To reduce the error caused by human factors or crop growth factors in judging the peak or outbreak period of the insect population, we objectively judged the peak period of insect occurrence by the third method. The seasonal activity curves were standardized following the method of Fazekas et al. (1997). The activity period of *B. tabaci* was divided into four quartiles based on the dates of 25, 50, and 75% of the total recorded individuals of whitefly. The proportion of the number of whiteflies in different sampling periods to the total number of sampling in the whole occurrence period is set as R. The period of the sampling population number of whitefly when R < 25% was defined as the “early activity period,” the period between 25% ≤ R < 75% is defined as the “main activity period,” and the period of 75% ≤ R ≤ 100% is defined as the “late activity period.” We define the date when *R* = 50% as the population peak during the whole occurrence period (notice that this is not linked to a density peak observed at a given time, it is a product of the cumulative curve, so it can also fall on a date when no census was carried out) (Fazekas et al., 1997; Zhang et al., 2014b).

### Landscape analysis

To define our study regions based on the landscape, an area with a radius of 0.5 km centered on the sampling field was demarcated using open-access satellite imagery from Google Earth and, combined with the change of land cover area in the selected area once a month. The principal component analysis method (PCA) (SPSS 20.0) was used to calculate the change factor in land cover with the greatest impact on insect population, determined as landscape type. We ignored features that were smaller than 5 m^2^ and could not be located during ground verification; the combined area of all unidentified fields was less than 0.1% of each landscape (Liu et al., 2016).

Landscape factors affecting insect populations by a change in land cover are classified into 10 categories: flowers, water, mountains, urban, vegetables, fruit trees, forest timbers, shrubs, grasslands, and wastelands. In all the landscapes sampling, we use the principal component analysis (PCA) method to calculate the eigenvalues and the cumulative proportion of the correlation matrix according to the correlation between variables in the landscape. Based on the results of the PCA, the first three principal components with cumulative contribution rates of > 80% were selected. According to the absolute value of the eigenvalues of different elements in the first principal component, the landscape was classified into four categories with the highest eigenvalue: urban landscape, flower landscape, water landscape, and mountain landscape (Tables 2, 3). Each landscape includes three replicates plots in different regions. The urban included buildings, roads, abandoned land, and other impervious surfaces. The flower included flowering plants such as *Rosa chinensis* Jacq. (Rosales: Rosaceae), *Dianthus caryophyllus* L. (Centrospermae: Caryophyllaceae), *Myosotis sylvatica* F. W. Schmidt (Tubiflorae: Boraginaceae), *Eustoma grandiflorum* (Raf.) Shinners (Gentianales: Gentianaceae), etc. The water included the river, the water channel, the pond, and the reservoir. The mountain included a forest with an altitude difference of more than 150 m.

**TABLE 2:** Eigenvalues of the covariance matrix and cumulative proportion of principal components at each agriculture landscape in 2018.

Land scapes	PC number	Eigenvalues										Cumulative proportion (%)
		
		Flowers	Water	Mountains	Urban	Vegetables	Fruit trees	Forest timbers	Shrubs	Grass lands	Waste lands	
1	PC1	0.9628	0.2341	0.0374	0.7815	–0.6686	0.3243	–0.4031	–0.4062	0.7966	–0.8615	38.50
	PC2	0.1950	0.5650	–0.7184	–0.2637	0.6653	0.8207	–0.7339	0.7734	–0.3794	–0.4784	74.19
	PC3	0.1597	0.7541	0.6574	–0.5221	0.3209	0.4334	0.4697	–0.4715	0.0630	–0.0868	94.63
2	PC1	0.9504	–0.3066	–0.5540	0.8677	0.5794	0.0413	0.6064	–0.3845	–0.0613	–0.8517	36.39
	PC2	0.2645	–0.5078	0.7964	0.2515	–0.7162	0.0780	0.5015	–0.8312	0.8607	0.4031	70.29
	PC3	0.1360	0.5478	0.1704	0.3361	–0.3080	–0.9219	0.4258	0.0392	–0.4754	0.2516	89.07
3	PC1	–0.9727	–0.8233	–0.6392	0.5151	0.8555	0.8934	0.7027	0.2368	–0.8317	0.3386	51.84
	PC2	–0.1624	–0.4273	0.5206	0.7032	0.4144	–0.4277	–0.4819	0.6175	0.3990	0.0374	72.87
	PC3	0.0949	–0.0006	–0.3847	–0.4074	0.2605	0.1268	0.2339	0.7226	0.3720	–0.9050	92.29
4	PC1	0.7737	–0.9561	0.4234	0.3566	–0.8188	0.7143	0.2931	0.4788	–0.8337	0.2003	40.50
	PC2	0.3954	0.2891	0.7550	0.2581	0.1760	–0.2456	0.7906	–0.8274	0.1455	0.8194	70.20
	PC3	0.3899	–0.0429	0.3356	0.8633	–0.0398	–0.6422	–0.4578	0.2742	0.3677	–0.2849	89.47
5	PC1	0.7811	–0.9920	–0.4408	–0.4612	–0.6224	0.7974	0.7375	0.3839	–0.4821	0.8962	47.51
	PC2	–0.3095	–0.0768	–0.7803	0.7873	–0.0489	0.1872	0.3489	0.8652	0.8685	–0.1849	77.78
	PC3	0.4129	–0.0497	0.2870	0.2177	0.7624	0.5591	0.4559	–0.2334	0.1004	–0.3507	93.70
6	PC1	0.6085	–0.9602	–0.6701	0.4755	0.8769	0.9023	0.7256	0.3015	–0.7927	–0.2030	48.38
	PC2	0.3044	–0.0210	0.2112	0.3096	0.4252	–0.3371	–0.3604	0.9154	0.5630	–0.7497	72.12
	PC3	0.4898	–0.2519	0.5774	0.7801	0.0885	–0.2677	–0.3793	–0.2511	–0.1487	0.6254	91.57
7	PC1	–0.4274	–0.4315	–0.9450	0.7809	0.8547	0.8778	–0.0561	0.2702	0.2895	–0.5077	37.91
	PC2	–0.0723	0.8637	0.2957	0.1646	0.0627	0.1103	0.9105	0.6750	–0.5825	–0.7477	68.55
	PC3	0.8933	–0.2392	0.0524	0.3865	–0.4483	–0.1428	0.0146	0.4458	0.6836	–0.4279	89.34
8	PC1	0.6370	0.8196	0.9658	–0.3127	0.4873	0.1804	–0.4586	0.8657	–0.1018	–0.4909	35.89
	PC2	–0.1082	0.0361	0.0223	0.7168	0.7313	–0.0084	0.4059	0.3746	–0.9883	0.7166	64.47
	PC3	–0.4084	0.1538	–0.1948	0.6140	0.2544	0.9779	–0.3407	–0.1983	0.1020	–0.4882	84.77
9	PC1	0.6561	–0.8660	–0.9680	0.8718	0.8298	0.2845	0.2777	0.1259	–0.7245	0.7674	48.54
	PC2	0.7181	0.4721	–0.1279	0.4696	0.0306	–0.7974	–0.8606	–0.5428	0.6861	0.5347	82.58
	PC3	0.1982	0.1012	0.2082	0.1073	0.4612	–0.4031	–0.0450	0.7720	0.0259	–0.3497	94.58
10	PC1	0.7390	0.8906	–0.5199	0.9463	–0.5168	–0.6838	0.8266	0.6274	–0.3405	–0.8719	51.93
	PC2	0.6463	–0.1577	–0.8340	0.1278	0.5526	0.4039	–0.0254	–0.6424	0.6513	–0.3623	77.85
	PC3	–0.1574	–0.3242	–0.0273	0.0669	0.6440	0.5965	0.3977	0.4270	–0.6169	–0.3001	95.02
11	PC1	0.2364	–0.5971	0.7613	–0.9062	0.7204	–0.7997	0.8277	–0.8651	–0.2225	0.8877	52.43
	PC2	0.6574	–0.6050	0.3205	0.3108	0.5772	0.2365	–0.3778	0.2977	–0.9603	–0.3933	79.38
	PC3	0.6121	0.5265	0.3457	–0.2867	–0.3344	0.4285	0.4147	0.4029	–0.1201	0.2352	94.91
12	PC1	0.0159	0.7759	0.7724	0.9541	–0.5746	0.4292	–0.7715	0.7884	0.7278	0.1244	43.86
	PC2	0.8565	0.4976	0.3615	–0.2113	0.7763	–0.6941	0.3197	0.1849	0.6651	–0.9376	80.84
	PC3	0.2568	–0.1260	0.4702	0.2047	–0.2588	0.5572	0.5075	–0.5381	–0.0060	–0.2612	94.22

PC, principal component.

**TABLE 3:** Eigenvalues of the covariance matrix and cumulative proportion of principal components at each agriculture landscape in 2019.

Land scapes	PC number	Eigenvalues										Cumulative proportion (%)
		
		Flowers	Water	Mountains	Urban	Vegetables	Fruit trees	Forest timbers	Shrubs	Grass lands	Waste lands	
1	PC1	0.9396	0.1618	0.6405	0.5987	–0.1358	0.8726	0.8678	0.7358	–0.7341	–0.8432	50.02
	PC2	–0.2874	–0.6518	0.5072	0.4171	0.9461	–0.1818	0.4965	–0.4110	0.6203	–0.4922	79.11
	PC3	0.1853	0.7220	0.5687	–0.3038	0.2891	–0.4480	0.0174	–0.3005	–0.2098	–0.0104	93.02
2	PC1	0.9845	0.4036	0.2496	0.4903	–0.3990	–0.2160	0.6751	0.7567	–0.7435	–0.7903	38.46
	PC2	–0.0022	–0.7238	–0.3867	0.5293	0.7646	0.3339	0.7230	–0.0021	0.5592	–0.5539	66.39
	PC3	–0.1738	–0.3563	0.8328	0.6351	–0.2235	0.8458	–0.0566	–0.1715	–0.3549	0.2614	88.85
3	PC1	0.9135	–0.3260	0.3433	0.0663	0.2399	–0.7998	0.8910	0.5684	0.7402	–0.8361	41.24
	PC2	–0.0427	0.4696	–0.7212	0.9157	0.7554	0.5617	–0.1670	–0.1472	0.6075	–0.5140	72.74
	PC3	0.3939	–0.8145	0.5507	0.3963	0.4622	0.1649	–0.4000	–0.4441	–0.0695	0.1907	91.92
4	PC1	0.8679	–0.9698	0.8155	–0.7040	–0.4752	0.7343	0.6161	0.7611	–0.7514	0.8766	59.11
	PC2	0.2317	–0.0602	–0.3752	0.6317	0.3484	0.6637	0.6873	0.6361	0.5872	–0.3387	84.07
	PC3	0.2765	0.1943	0.3725	–0.1140	0.8071	0.0267	–0.1526	0.0648	–0.2902	–0.2793	95.15
5	PC1	0.8917	–0.9922	–0.6306	–0.5428	–0.4954	0.7961	0.6787	0.8438	–0.6223	0.8637	56.57
	PC2	0.1257	0.0915	–0.2786	0.7371	0.4580	0.5386	0.5614	0.5200	0.6748	–0.4615	80.56
	PC3	0.2186	0.0819	0.6058	–0.3490	0.6153	0.2010	0.1241	0.1237	–0.3335	–0.1996	92.00
6	PC1	–0.6102	0.9294	0.7118	0.7586	–0.8543	0.3059	0.1750	0.0351	0.4955	–0.8867	42.05
	PC2	0.3812	0.3308	0.0933	–0.3978	0.3126	0.9365	0.7933	–0.6852	–0.2826	–0.1875	68.16
	PC3	0.5848	–0.0929	–0.0114	0.4885	–0.1720	–0.1438	–0.0254	–0.7120	0.7168	0.3923	86.31
7	PC1	–0.3860	0.3172	0.9152	0.8603	0.6878	0.4996	0.2212	0.3258	–0.7167	–0.8579	39.54
	PC2	0.8669	0.5099	–0.3825	–0.2560	0.6170	0.7272	–0.6726	0.7039	–0.1541	0.2744	71.34
	PC3	–0.1337	0.6519	0.0492	–0.4182	–0.2262	–0.3118	0.6230	0.6274	–0.1228	0.0730	87.05
8	PC1	–0.4530	–0.8439	0.9001	0.6950	0.4853	0.4556	0.6579	0.3544	–0.8558	–0.7141	44.54
	PC2	0.8886	–0.4279	–0.4157	–0.2134	0.6356	0.6847	–0.3514	0.6673	–0.4622	0.6404	77.11
	PC3	0.0716	0.3084	0.0346	0.6374	0.5986	–0.3858	–0.6535	–0.1467	–0.1117	–0.1264	92.03
9	PC1	0.3449	–0.4970	0.9177	0.2084	0.8087	0.7346	0.2192	–0.5511	–0.1746	–0.8788	36.00
	PC2	–0.9262	–0.8370	–0.2141	0.7026	0.3983	0.0812	–0.2710	0.2865	0.9817	0.0450	69.84
	PC3	0.0325	0.0768	0.0955	0.6715	–0.4023	–0.4141	0.9279	0.1728	0.0568	–0.3763	88.20
10	PC1	–0.3811	–0.5972	0.6601	–0.9460	0.6930	–0.8760	0.7928	–0.7530	0.8865	0.9440	59.53
	PC2	0.8528	–0.3633	0.6254	–0.1014	0.4786	0.3124	0.0189	0.4728	–0.1097	–0.0216	77.76
	PC3	0.2632	0.7118	0.1912	–0.3076	–0.5247	0.1881	0.6091	0.1369	–0.0605	0.3289	92.96
11	PC1	0.2637	0.8645	–0.6746	0.9108	–0.6599	0.8602	–0.8311	0.7387	–0.3187	–0.8873	54.02
	PC2	0.8842	0.0625	0.6805	–0.2432	0.0637	0.5024	0.3155	0.2205	–0.9246	0.2164	80.16
	PC3	–0.0060	–0.4843	0.2858	0.3213	0.7486	0.0258	–0.4433	0.0635	–0.0762	–0.3975	93.61
12	PC1	0.5250	–0.4654	0.8672	0.9792	–0.6318	0.2787	0.3840	–0.8680	0.4429	–0.8964	45.80
	PC2	–0.2407	0.1459	0.3022	0.0024	0.1802	0.9217	–0.8430	0.3191	0.3014	–0.2832	66.17
	PC3	0.8133	0.2456	0.0110	0.1940	0.7500	0.2387	0.3448	0.3769	–0.3532	–0.2747	84.57

PC, principal component.

### Statistical analysis

The number of each species in the community obtained from each leaf survey was used as the basis for the data analysis. This value was used to calculate the dominance of each species and to screen dominant predators in different agricultural landscapes. The dominant species was selected for the temporal niche analysis. Dominance was expressed as relative density, and species with dominance greater than 10% were denoted dominant species (Gao et al., 2014).


$$\text{Dominance}=\frac{\mathrm{The}\,\mathrm{number}\,\mathrm{of}\,\mathrm{individuals}\,\mathrm{of}\,a\,\mathrm{species}}{\text{The}\,\text{total}\,\mathrm{number}\,\mathrm{of}\,\mathrm{individuals}\,\mathrm{of}\,\mathrm{the}\,\mathrm{species}}$$

The calculation of the temporal niche correlation index was based on Levins and Hurlbert formula (Levins, 1969; Hurlbert, 1978).

Temporal niche width:


$$B\,i=\frac{1/\sum_{i=1}^{n}p_{i}^{2}-1}{S-1}$$

where *P*_*i*_ is the proportion of species in unit *i* in a resource set and *S* is the total number of units in the resource set.

Temporal niche overlap:


$$a_{i\,j}=\sum_{h=1}^{n}P_{i\,h}\,P_{j\,h}\,\left(B_{i}\right)$$

where *P*_*ih*_ and *P*_*jh*_ represent the proportions of species *i* and *j* in unit *h*. *B*_*i*_ is the width of the temporal niche of species *i*.

### Data analysis

The census data were initially divided by the quartile method, and the population density of the main activity period was analyzed using one-way ANOVA (repeated measures) after tests of normality (Shapiro–Wilk) and homoscedasticity (Bartlett), while densities during the early activity period and the late activity period were excluded because they were too low to distinguish a difference between the treatments. Differences in *B. tabaci* and its predator densities were compared between agricultural landscapes on the same dates, as well as between weeks of each agricultural landscape, restricted by season, Tukey’s HSD (*P* = 0.05). A significance level of *P* = 0.05 was used for all tests. Data analyzes were performed using SPSS 20.0 (Zhang et al., 2014b). The cumulative seasonal activity curves and population dynamics were made using Origin 2018.

## Results

### Seasonal activity of *Bemisia tabaci*

**FIGURE 1:**
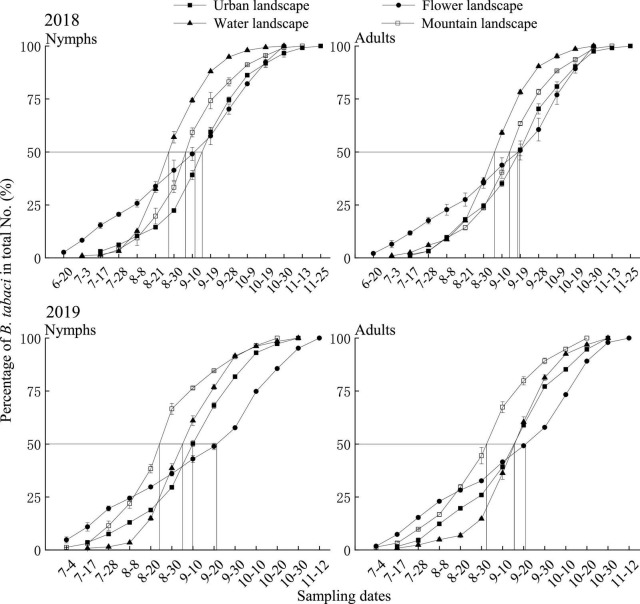
Cumulative seasonal activity curves of *Bemisia tabaci* on summer tomato fields in different agricultural landscapes in Kunming city, Yunnan province, South China, in 2018 and 2019. Urban landscape: Tomato fields of an agricultural landscape dominated by the urban area, flower landscape: tomato fields of an agricultural landscape dominated by flowers, water landscape: tomato fields of an agricultural landscape dominated by water, and mountain landscape: tomato fields of an agricultural landscape dominated by mountains. The same is applicable to Figures 2, 3.

In 2018, the length of the main activity period of *B. tabaci* nymphs was 21 days in the water landscape, 29 days in the mountain and urban landscape, and 63 days in the flower landscape. Peak activity was recorded between August 27 in the water landscape and September 15 in the urban landscape (Table 4). For adults of *B. tabaci*, the main activity period lasted 29 days in the urban landscape, 30 days in the water landscape, 32 days in the mountain landscape, and 52 days in the flower landscape. The earliest seasonal activity peak occurred on September 6 in the water landscape and the latest on September 18 in the urban landscape (Table 4 and Figure 1).

**TABLE 4:** The main activity period and dates of peak activity of nymph and adult *Bemisia tabaci* on summer tomato fields in different agricultural landscapes.

Landscape patterns	Years	Nymphs		Adults	
			
		Main activity period (duration in days)	Peak activity date	Main activity period (duration in days)	Peak activity date
Urban	2018	8–30 to 9–28 (29)	9–15	8–30 to 9–28 (29)	9–18
	2019	8–30 to 9–30 (32)	9–10	8–30 to 9–30 (32)	9–15
Flower	2018	7–28 to 9–28 (63)	9–12	8–8 to 9–28 (52)	9–17
	2019	8–20 to 10–10 (52)	9–21	8–20 to 10–10 (52)	9–21
Water	2018	8–21 to 9–10 (21)	8–27	8–21 to 9–19 (30)	9–6
	2019	8–30 to 9–20 (22)	9–5	9–10 to 9–30 (21)	9–15
Mountain	2018	8–20 to 9–17 (29)	9–6	8–29 to 9–29 (32)	9–14
	2019	8–8 to 9–10 (34)	8–24	8–20 to 9–20 (32)	9–3

In 2019, the main activity period of the nymphs ranged from 22 days in water landscapes to 52 days in the flower landscape, and the activity peaked on August 24 in the mountain landscape and September 21 in the flower landscape (Table 4). For adults, the main activity period lasted 21 days in the water landscape, 32 days in the urban and mountain landscapes, and 52 days in the flower landscape (Table 4). The earliest seasonal activity peak was in the mountain landscape on September 3 and the latest in the flower landscape on September 21 (Table 4 and Figure 1).

### Seasonal population dynamics of *Bemisia tabaci*

**FIGURE 2:**
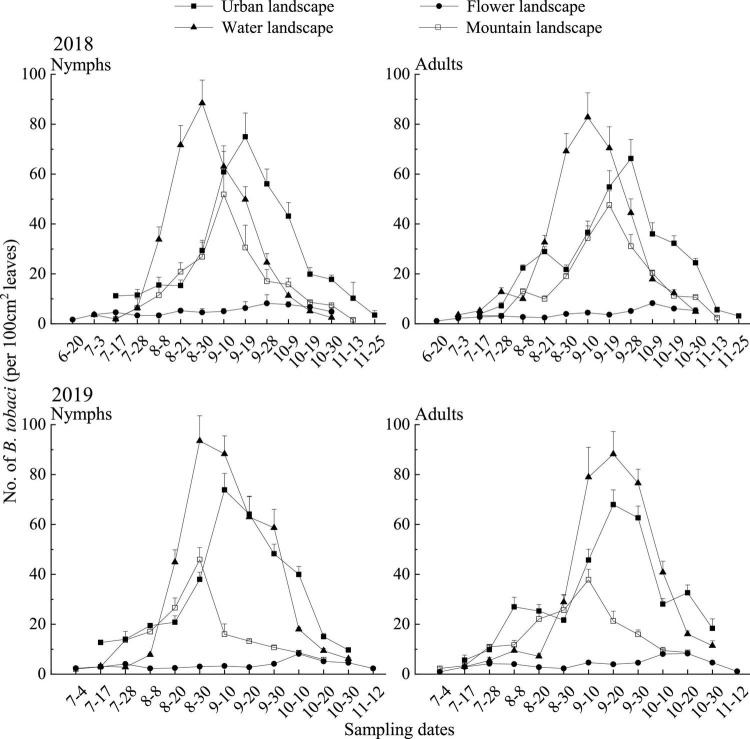
Seasonal dynamics of *Bemisia tabaci* (mean + SE) on summer tomato fields in different agricultural landscapes at Kunming city, Yunnan province, South China, in 2018 and 2019.

In 2018, the densities of *B. tabaci* nymphs in the water landscape exceeded the values observed in the other landscapes throughout the sampling season, with the highest densities (88.41 individuals per 100 cm^2^ leaves) being recorded on August 30, about 10–20 days earlier than in other landscapes (Figure 2). During the main activity period, the densities of whitefly nymph in the water landscape were the highest (74.36 individuals per 100 cm^2^ leaves), which was significantly higher than the other three landscapes [*F*_(3, 50)_ = 79.24; *P* = 0.0001]. After the water landscape, the urban landscape supported the highest densities of nymphs, and the densities of nymphs have the lowest in the flower landscape (4.85 individuals per 100 cm^2^ leaves) (Table 5). *Bemisia tabaci* adult population was first observed on June 20 and continued to increase until the population peaked in September, followed by a gradual decrease until the end of the survey. The densities in the water landscape were significantly [*F*_(3, 50)_ = 44.52; *P* = 0.0001] higher than in the other three landscapes during the main activity period (Table 5). The highest density in the water landscape was observed on September 10 as 82.77 adults per 100 cm^2^ leaves (Figure 2).

**TABLE 5:** Population density of *Bemisia tabaci* and its dominant predators during the main activity period on summer tomato fields in four different agricultural landscapes.

Landscape patterns	Years	Population density (per 100 cm^2^ leaves)					
		
		*Bemisia tabaci*		*Nesidiocoris tenuis*	*Chrysoperla sinica*	*Menochilus sexmaculata*	*Harmonia axyridis*
		
		Nymphs	Adults				
Urban	2018	55.29 ± 5.47b	44.86 ± 5.45b	0.52 ± 0.02b	0.29 ± 0.02b	0.33 ± 0.02b	−
Flower	2018	4.85 ± 0.48d	3.71 ± 0.33c	0.65 ± 0.02a	0.44 ± 0.02a	0.47 ± 0.02a	0.49 ± 0.02a
Water	2018	74.36 ± 5.02a	63.73 ± 6.15a	0.39 ± 0.02c	0.29 ± 0.01b	0.34 ± 0.02b	−
Mountain	2018	32.49 ± 4.02c	33.02 ± 3.60b	0.49 ± 0.02b	0.37 ± 0.02a	0.28 ± 0.02b	0.26 ± 0.01b
Urban	2019	56.05 ± 4.70b	49.47 ± 5.63b	0.67 ± 0.02a	0.25 ± 0.02c	0.36 ± 0.02b	−
Flower	2019	3.97 ± 0.49d	4.37 ± 0.46d	0.66 ± 0.03a	0.44 ± 0.02a	0.56 ± 0.04a	0.36 ± 0.02b
Water	2019	81.57 ± 5.78a	81.24 ± 3.51a	0.43 ± 0.03b	0.33 ± 0.02bc	0.42 ± 0.04b	−
Mountain	2019	26.39 ± 3.83c	26.73 ± 2.77c	0.47 ± 0.02b	0.39 ± 0.02ab	0.35 ± 0.02b	0.49 ± 0.02a

The value is the mean ± SE, Different lowercase letters indicate significant differences between different landscapes of the same insect in the same year (P < 0.05).

In 2019, the water landscape had the highest densities of whitefly nymphs during the main activity period (81.57 individuals per 100 cm^2^ leaves), significantly higher than the other three landscapes [*F*_(3, 50)_ = 91.99; *P* = 0.0001], followed by the urban landscape (Table 5). The general trend of the seasonal dynamic curve of adults was similar to that observed in 2018. Adults of whitefly were first observed on July 4, followed by a steady increase until mid-September (Figure 2). During the main activity period, adult densities were the highest in the water landscape (81.24 individuals per 100 cm^2^ leaves), and significantly higher than in the other three landscapes [*F*_(3, 50)_ = 100.29; *P* = 0.0001; Table 5].

### Species and dominance of predators of *Bemisia tabaci*

Nine predator species, belonging to four orders and five families, were sampled in different agricultural landscapes of summer tomato fields. *Nesidiocoris tenuis* (Reuter) (Hemiptera: Miridae), *Chrysoperla sinica* Tjeder (Neuroptera: Chrysopidae), and *Menochilus sexmaculata* (Fabricius) (Coleoptera: Coccinellidae) were the dominant species in urban and water landscape tomato fields, while *N. tenuis*, *C. sinica*, *M. sexmaculata*, and *Harmonia axyridis* Pallas (Coleoptera: Coccinellidae) were the dominant species in the flower and mountain landscapes, both in 2018 and 2019 (Table 6).

**TABLE 6:** Species and dominance of the predators of *Bemisia tabaci* on summer tomato fields in different agricultural landscapes.

				Dominance (%)			
				
Order	Family	Species	Years	Landscape patterns			
				
				Urban	Flower	Water	Mountain
Hemiptera	Miridae	*Nesidiocoris tenuis*	2018	33.73 ± 1.03a	25.68 ± 0.42b	22.32 ± 1.39b	22.2 ± 0.95b
			2019	41.25 ± 1.11a	27.30 ± 0.54b	21.85 ± 1.39c	20.27 ± 0.35c
Neuroptera	Chrysopidae	*Chrysoperla sinica*	2018	17.16 ± 0.73a	14.50 ± 0.24a	16.19 ± 0.95a	15.82 ± 0.95a
			2019	13.46 ± 0.42a	13.14 ± 1.05a	12.32 ± 0.43a	15.16 ± 0.33a
Coleoptera	Coccinellidae	*Menochilus sexmaculata*	2018	21.59 ± 0.31a	17.05 ± 0.16b	20.89 ± 0.93a	12.61 ± 0.38c
			2019	18.87 ± 0.89ab	17.82 ± 0.23ab	23.36 ± 2.30a	13.13 ± 0.46b
		*Harmonia axyridis*	2018	5.74 ± 0.94c	16.27 ± 0.43a	8.75 ± 1.09bc	11.01 ± 0.34c
			2019	6.44 ± 0.80c	10.69 ± 0.44b	7.94 ± 0.89bc	20.59 ± 0.71a
		*Lemnia biplagiata*	2018	0.00 ± 0.00b	6.89 ± 0.86a	8.57 ± 0.65a	6.39 ± 0.63a
			2019	0.00 ± 0.00c	4.11 ± 0.21b	8.17 ± 1.18a	4.77 ± 0.31b
		*Coccinella septempunctata*	2018	4.75 ± 0.78b	3.00 ± 0.34bc	0.00 ± 0.00d	8.19 ± 0.40a
			2019	4.12 ± 0.42a	4.86 ± 1.06a	0.00 ± 0.00b	4.77 ± 0.20a
		*Propylaea japonica*	2018	0.00 ± 0.00d	3.80 ± 0.61bc	5.17 ± 0.54ab	6.818 ± 0.43a
			2019	0.00 ± 0.00a	3.40 ± 0.43a	8.02 ± 0.99a	8.35 ± 0.47a
Araneida	Linyphiidae	*Hylyphantes graminicola*	2018	8.73 ± 0.40a	6.59 ± 0.69a	9.12 ± 0.68a	9.17 ± 1.05a
			2019	9.65 ± 1.38a	8.63 ± 0.61a	9.27 ± 0.83a	7.34 ± 0.52a
	Theridiidae	*Theridion octomaculatum*	2018	8.31 ± 1.09a	6.23 ± 0.46a	8.93 ± 1.07a	7.99 ± 0.39a
			2019	6.21 ± 0.72bc	9.92 ± 0.74a	9.06 ± 0.92ab	5.62 ± 0.31c

The dominance value is the mean ± SE, different lowercase letters indicate significant differences between different landscapes of the same insect in the same year (P < 0.05).

### Population dynamics of dominant predators of *Bemisia tabaci*

**FIGURE 3:**
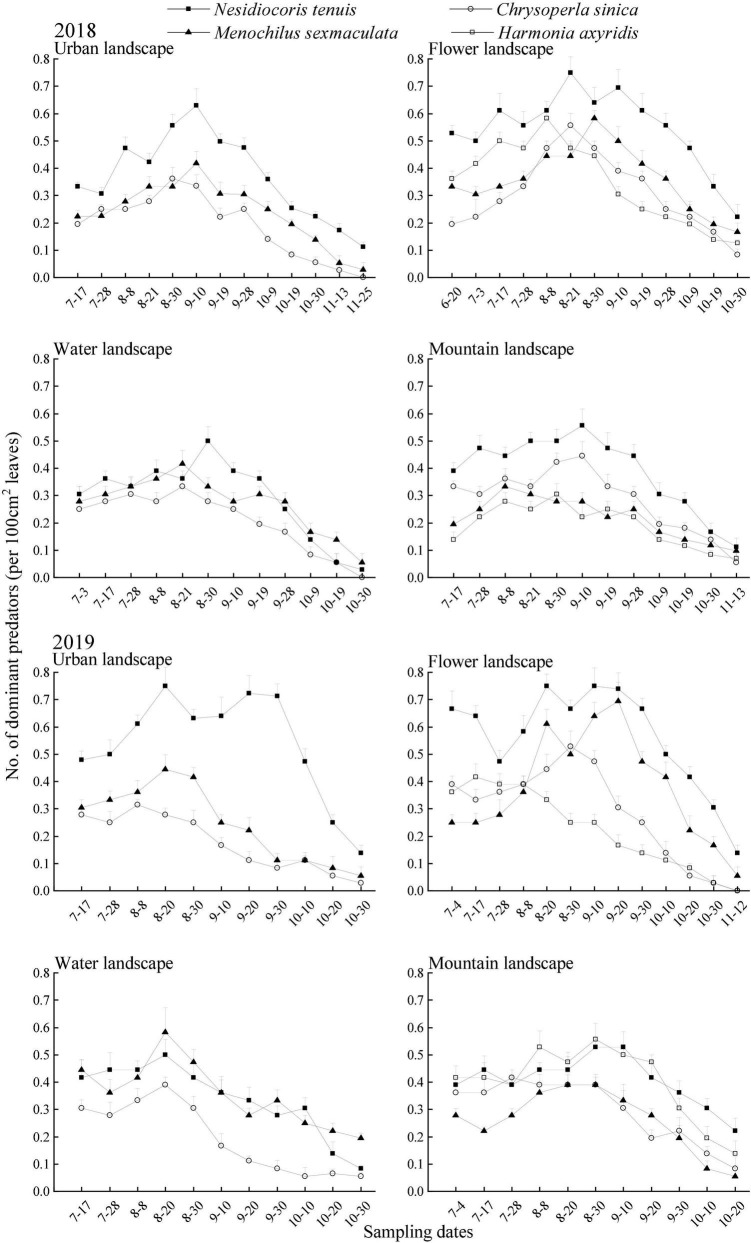
Seasonal dynamics of the dominant predators of *Bemisia tabaci* (mean + SE) on summer tomato fields in different agricultural landscapes at Kunming city, Yunnan province, South China, in 2018 and 2019.

In the 2 years of surveys, each reported predatory had a small number of populations at the beginning of the investigation. Thereafter, continued to grow. In 2018, the predator population peaks appeared in urban and mountain landscapes in early September, and predator population peaks appeared in flower and water landscapes in the middle and late August. In 2019, the population peaks of predators appeared in urban and water landscapes in mid-August and the population peaks of predators in flower and mountain landscapes appeared in late August. The population densities of the predators decreased continuously after peaking until the end of the survey during both years of surveys (Figure 3).

In 2018, during the main activity period of the predators, the population density of the four dominant predators was the highest in the flower landscape, and the population density of *N. tenuis* is the highest (0.65 individuals per 100 cm^2^ leaves; Table 5). In 2019, during the main activity period of the predators, the population density of *N. tenuis* in the urban landscape was the highest (0.67 individuals per 100 cm^2^ leaves), and that in the flower landscape was the second (0.67 individuals per 100 cm^2^ leaves), without significant differences [*F*_(3, 56)_ = 24.45; *P* = 0.99]. Both *C. sinica* and *M. sexmaculata* showed the highest population density in the flower landscape, while *H. axyridis* showed the highest population density in the mountain landscape (0.49 individuals per 100 cm^2^ leaves; Table 5).

### Temporal niche analysis of *Bemisia tabaci* and its dominant predators

Based on the temporal niche widths of *B. tabaci* and its dominant predators, the two main temporal niche widths in the four different agricultural landscapes were shown by *N. tenuis* and *M. sexmaculata* in 2018, while in 2019, the highest temporal niche width was shown by *N. tenuis*. During the study period of 2 years, each natural enemy had the largest width of the niche in the flower landscape. In the water landscape, the population density of whitefly was the largest, while the temporal niche width was the lowest compared to that in the other three agricultural landscapes (Table 7). Among the four different agricultural landscapes, the temporal niche overlap index of *B. tabaci* and *N. tenuis* was highest in 2018, while that of *B. tabaci*, *N. tenuis*, and *M. sexmaculata* was highest in 2019 (Table 7).

**TABLE 7:** Temporal niche parameters of *Bemisia tabaci* and its dominant predators on summer tomato fields in four different agricultural landscapes.

Species	Years	*B. tabaci*				*N. tenuis*				*C. sinica*				*M. sexmaculata*				*H. axyridis*	
						
		Urban	Flower	Water	Mountain	Urban	Flower	Water	Mountain	Urban	Flower	Water	Mountain	Urban	Flower	Water	Mountain	Flower	Mountain
*B. tabaci*	2018	8.14	11.51	6.12	7.13	0.95	0.96	0.88	0.93	0.91	0.93	0.85	0.93	0.95	0.95	0.86	0.92	0.91	0.93
	2019	7.70	10.97	5.90	6.67	0.95	0.94	0.83	0.92	0.86	0.83	0.77	0.91	0.87	0.92	0.85	0.93	0.84	0.92
*N. tenuis*	2018					11.14	12.23	9.77	10.70	0.97	0.99	0.99	1.00	0.99	1.00	0.99	1.00	0.98	1.00
	2019					9.82	11.79	9.68	10.53	0.96	0.96	0.97	0.99	0.97	0.99	1.00	0.98	0.95	0.99
*C. sinica*	2018									9.58	10.97	9.60	10.38	0.98	0.99	0.98	0.99	0.98	0.99
	2019									8.50	9.60	7.85	9.65	0.99	0.95	0.98	0.99	0.98	0.99
*M. sexmaculata*	2018													10.78	11.83	10.62	10.79	0.98	1.00
	2019													8.52	10.40	9.36	9.38	0.91	1.00
*H. axyridis*	2018																	11.08	10.40
	2019																	9.35	9.97

Values in the main diagonal are niche width parameters and values on the main diagonal are niche overlap parameters.

## Discussion

The structure composition of the agricultural landscape is an important factor that affects the occurrence, density, and dynamics of the insect population (Bianchi et al., 2006). The results indicated significant differences in the peak period and population density of *B. tabaci* and its predators in tomato fields in four different agricultural landscapes. During both years, the water landscape supported the highest density of whiteflies (nymphs and adults). The population density of the nymph and adult of the whitefly was the lowest in the flower landscape. In the study of the population dynamics of whiteflies in different agricultural planting environments, Dou et al. (2020b) also found that the population density of whiteflies in the flower agricultural planting environment was the lowest, which was consistent with the results of our study. We hypothesize that this result is affected by both the composition of the crop and predators in the landscape. The resource concentration hypothesis suggests that phytophagous insects prefer to live in a single host plant habitat (Zhao Z. H. et al., 2015). In this study, the other crops planted around the tomato field in the water landscape were mainly *Nelumbo nucifera* Gaertn (Proteales: Nelumbonaceae), *Vitis vinifera* L. (Rhamnales: Vitaceae), and *Allium tuberosum* L. (Liliflorae: Liliaceae). The diversity of vegetation was low, and they were not the preferred host plant of whiteflies compared to tomatoes (Heng et al., 2017). The proportion of man-made construction land in the urban landscape was also large, and the host plants of the whitefly were relatively single. Therefore, the population density of the whitefly is lower in the early stage of occurrence in the water landscape and urban landscape, and the outbreak is concentrated in tomato fields during the main occurrence period. The number and damage of pests in intercropping of different crops are reduced to varying degrees compared to a single planting (Thies and Tscharntke, 1999). Studies have shown that the intercropping of *S. lycopersicum–Apium graveolens* L. (Apiales: Apiaceae) or *C. sativus–A. graveolens* can effectively control the occurrence of *Trialeurodes vaporariorum* (Westwood) (Hemiptera: Aleyrodidae) (Zhu et al., 2011). Intercropping of main crops with trap plants can be beneficial. Planting *Solanum melongena* in main *Phaseolus vulgaris* L. (Rosales: Fabaceae) fields can attract whitefly on *P. vulgaris* and greatly reduce the population (Smith and Mcsorley, 2000). A study by Russell (1989) demonstrated that the number of pest insects in a single-crop planting system was significantly higher than in a diversified system. Yang et al. (2021) research showed that the population density of whitefly in tomato monoculture fields was higher than in other adjacent cropping patterns during the main occurrence period, which was similar to the findings of our study.

This study shows that the population density of predators was different among the four landscapes, resulting in significant differences in the density of whiteflies. In the flower landscape, the species of dominant predators of whiteflies showed the highest richness and diversity. Studies have shown that landscape factors are the key drivers of predator abundance (Woltz et al., 2012). The natural enemy hypothesis suggests that diverse plant communities can increase natural enemy populations (Zhao Z. H. et al., 2015). Flowering plants can significantly increase the life span of natural enemies and the number of their eggs (Lisa and Steve, 2004). Nectar plants are abundant in the flower landscape. It provides nectar, pollen, the activity place for habitat and reproduction of natural enemy insects (Petanidou et al., 2006; Borghi and Alisdair, 2017), resulting in the highest population density of predators of whitefly in flower landscape tomato fields and inhibiting the outbreak of the whitefly population. This is similar to the research results of Johanowicz and Mitchell (2000), in which the plantation of flowering plants around economic crops had a positive effect on pest control. Moreover, crop diversity can significantly increase the number of natural enemy insects (Dong et al., 2016). The population density of Coccinellidae can be significantly increased by intercropping *Saccharum officinarum* L. (Poales: Poaceae)-*Z. mays* (Zhang et al., 2011). The number of parasitoids in a mixed field of *Cucurbita moschata* (Duch. ex Poiret) (Cucurbitales: Cucurbitaceae)*-Z. mays–Pisum sativum* L. (Rosales: Fabaceae) is more than two times that in the *C. moschata* monoculture field (Letourneau, 1987). However, in this study, the diversity of host plant in the urban landscape and the water landscape with low, and the population density of *M. sexmaculata* is richer than in the mountain landscape, after the flower landscape. This may be due to the relatively large population density of whitefly in urban landscapes and water landscapes, and the strong predation ability of *M. sexmaculata*, with an obvious following effect (Wu et al., 2010). The population density of *N. tenuis* is the highest in four different agricultural landscapes, mainly because *N. tenuis* belongs to omnivorous insects and can suck plant juice when the pest population is low, so it is less affected by the pest population (Zhou et al., 2012). *H. axyridis* was abundant in the flower landscape in the first year, but in the second year much more was recorded in the mountain landscape. It may be due to the different populations of predators in different landscapes at the beginning of the sampling each year. There is a competitive or predatory relationship between two species with niche overlap (Li et al., 2006). From the perspective of niche, the competitive relationship between several pests and the control of pests by predators can be better analyzed (Dolédec et al., 2000). In this study, *B. tabaci, N. tenuis*, and *M. sexmaculata* were temporally synchronized, with high similarity in time resources. This result is consistent with the study by Li et al. (2021) on the population dynamics and temporal niches of whitefly and their dominant predators in cucumber and tomato fields. The values of the temporal niche width parameter of each species and the temporal niche overlap parameter of whitefly and its predators were the highest in flower landscapes. In the flower landscape, the encounter frequency between predators and whitefly at the same time is higher than that in the other landscapes, and it shows that the control effect of predators on whitefly is better (Hurlbert, 1978). Planting flowering plants of *Asteraceae* in winter wheat fields can significantly enhance the control effect of natural enemies on wheat *Chrysomelidae* pests (Tschumi et al., 2015, 2016). Planting flowering plants such as *Tagetes erecta* L. (Asterales: Asteraceae), *Callistephus chinensis* (L.) (Campanulales: Asteraceae), and *Medicago sativa* L. (Rosales: Fabaceae) in apple orchards can increase the control effect of natural enemy insects on *Grapholita molesta* Busck (Lepidoptera: Tortricidae) (Mu et al., 2019). These studies were consistent with our findings.

From the perspective of the vegetation diversity and the landscape complex, flower landscape and mountain landscape have more plant species than other landscapes in our study. Based on the ecological regulation theories of pests such as the landscape complexity hypothesis, using plant diversity for habitat regulation can effectively improve the colonization rate of natural enemies and the ability of sustainable pest control (Cook et al., 2007). Complex plant diversity represents more plant species, which can provide food, wintering, and breeding habitat for natural enemies and help them reduce the possibility of pesticides and farming interference. According to the role and function of natural enemies, it can be divided into banker plant, nectar resource plant, habitat plant, trap plant, indicator plant, and guardian plant (Parolin et al., 2012; Chen et al., 2014). They are conducive to the growth of natural enemy populations in the ecosystem (Chen et al., 2014; Miller et al., 2018). Moreover, complex plant diversity can also increase insect diversity within the ecosystem. Most predatory natural enemies are polyphagous insects. The increase in insect diversity also provides predatory natural enemies with more diverse alternative prey like aphids, thrips, mealybugs, etc., which is also conducive to the reproduction of natural enemy insects (Yongheon et al., 2004; Pineda and Marcos-García, 2008; Rattanapun, 2017).

The natural control ability of the whitefly predators was greatly affected by the change in the planting environment (Zhang et al., 2020a). The flower landscape has obvious advantages among the four agricultural landscapes. Flowering plants can provide nutrients such as pollen and nectar to predators (Zhu et al., 2012). However, herbivorous insects can also use pollen and nectar as food sources to increase their population (Heimpel and Jervis, 2006). Thus, it is necessary to select flowering plants and obtain favorable flower species with care. The principle is to select flowering plants that can maximize the benefits to predators and minimize the interest of pests during crop growth (Evans et al., 2010). In the water landscape, due to the single planting mode and low vegetation diversity, it is easy to cause an outbreak of the pest population, and the occurrence and damage of the tomato field by whitefly was the most serious in that landscape. Therefore, a suitable layout of the agricultural landscape, such as fixing an appropriate proportion of flowering plants and increasing the diversity of planting crops, can effectively reduce the possibility of a whitefly outbreak.

## Data availability statement

The raw data supporting the conclusions of this article will be made available by the authors, without undue reservation.

## Author contributions

G-HC, X-MZ, and W-JD designed the study. W-JD, M-JL, S-WY, and H-PS performed the experiments. M-JL, S-WY, and X-MZ analyzed the data and wrote the manuscript. All authors read and approved the final manuscript.
